# Good Enough to Sequence: Fluorometry-Guided Salvage of Low-Volume and Compromised Paediatric DNA in Custom Amplicon Next-Generation Sequencing

**DOI:** 10.3390/diagnostics16213447

**Published:** 2026-10-22

**Authors:** Prateek Bhatia, Sharun Sharun, Meenakshi Malhotra, Sangeetha Kirubanandhan, Parminder Kaur, Deepshikha Bhardwaj, Rozy Thakur, Minu Singh

**Affiliations:** Paediatric Molecular Haematology-Oncology Laboratory, Paediatric Haematology-Oncology Unit, Department of Paediatrics, Advanced Paediatrics Centre, Postgraduate Institute of Medical Education and Research (PGIMER), Chandigarh 160012, India; sharun4444@gmail.com (S.S.); meenakshimalhotra555@gmail.com (M.M.); sangeethahsr@gmail.com (S.K.); kaur2211parminder@gmail.com (P.K.); shikhabhrdwaj185@gmail.com (D.B.); rozythakur2512@gmail.com (R.T.); meenusingh.dr@gmail.com (M.S.)

**Keywords:** custom targeted NGS panel, Qubit, Nanoquant, paediatric samples, low-volume/compromised DNA, Bland–Altman, method agreement

## Abstract

**Introduction:** Amplicon-based next-generation sequencing (NGS) is widely used in clinical practice, yet few studies address the impact of fluorometric quality control and of low-volume or compromised paediatric samples on library quantification and sequencing metrics. We reframe this question around a practical goal: whether sub-optimal paediatric DNA can be reliably salvaged for custom-panel amplicon NGS. **Materials and Methods:** Data from 314 paediatric samples sequenced on 16 custom-designed amplicon panels (Ion Torrent S5) were analysed retrospectively by Qubit input quantity (Group A ≥ 10 ng/µL; Group B < 10 ng/µL) and Nanoquant A260/280 quality (Group C 1.7–1.9; Group D < 1.7 or >1.9), and compared against post-library quantification, mean depth, and ten coverage QC parameters. Method agreement (Bland–Altman, proportional-bias regression), effect sizes (Cliff’s δ), panel-adjusted mixed-effects models and ROC/logistic prediction of library failure were added. **Results:** Median input was 59.75 ng/µL by Nanoquant and 28.25 ng/µL by Qubit (*p* < 0.0001); median A260/280 was 1.86. Nanoquant overestimated DNA ~2.1-fold, but Bland–Altman analysis revealed wide, concentration-dependent limits of agreement (mean ratio 2.17×; 95% LoA 0.57–8.33×; proportional-bias *p* < 0.0001), indicating that no single correction factor is reliable. Group A comprised 91.7% (288/314) and Group B 8.3% (26/314); Group C 75.2% (236/314) and Group D 24.8% (78/314). Median post-library Qubit and mean depth were 1254 vs. 552 ng/mL (*p* < 0.0001) and 848× vs. 1352× (*p* = 0.025) for A vs. B, and 1254 vs. 1026 ng/mL (*p* = 0.049) and 745× vs. 1297× (*p* < 0.0001) for C vs. D. The higher depth in quantity Group B lost significance after adjusting for panel (mixed-model *p* = 0.083), identifying it as confounding. Input DNA predicted library failure (<300 ng/mL), with an ROC AUC of 0.79 and an exploratory empirical optimal threshold of ≈21 ng/µL (derived and tested in the same cohort; 14 failure events). None of the ten coverage QC parameters differed across groups. **Discussion and Conclusions:** Qubit quality control is strongly recommended because Nanoquant inflates readings ~2.1-fold, with no dependable correction factor. Low-volume or compromised paediatric DNA (<10 ng/µL and/or A260/280 outside 1.7–1.9) can be salvaged for amplicon NGS with defined protocol adaptations, with only ~19–23% of the worst-case samples failing library QC and comparable coverage in a small, concentration-matched representative subset (*n* = 5 per group) that was underpowered for formal equivalence testing. An empirical input threshold of ≈20 ng/µL, roughly double the manufacturer’s minimum, best protects against library failure.

## 1. Introduction

Amplicon panel-based next-generation sequencing (NGS) is a multiplex PCR approach generating amplicons for target regions by designing and multiplexing up to ~12,000 primer pairs in a 2- to 5-tube format. It allows rapid, cost-efficient generation of extensive genomic data for in-depth examination of inherited disease and cancer. The NGS workflow comprises four core steps: extraction and quantification of nucleic acids, library preparation, template generation and sequencing. Success depends on the quality and quantity of input material, batch quality, reagent stability and technical competence; among these, high-quality extraction and accurately quantified optimal input are the most critical. Available DNA quantification methods range from spectrophotometry to fluorometry, capillary gel electrophoresis, real-time PCR and droplet digital PCR (ddPCR) [1,2].

DNA purity is commonly assessed by the 260/280 nm absorbance ratio, with ~1.8 indicating relatively pure DNA. However, spectrophotometry has limited sensitivity (~1 ng/µL) and cannot discriminate between single-stranded DNA, double-stranded DNA, RNA and free nucleotides [3,4]. Quantitative PCR is sensitive and specific but laborious [5], while capillary electrophoresis and ddPCR carry higher capital and logistical costs [1,3,6]. Most NGS workflows therefore recommend dsDNA-specific fluorometric assays such as Qubit at the input and post-library steps. Nonetheless, in many resource-constrained laboratories, particularly in low- and middle-income countries, spectrophotometric methods (Nanoquant/NanoDrop) remain in routine use even for sensitive NGS or microarray assays.

Several studies show that spectrophotometry gives falsely high DNA values, 3- to 5-fold above fluorometry or capillary electrophoresis, although some report limited downstream effect on variant detection [7,8,9,10,11]. Having run amplicon sequencing for six years in a resource-constrained setting, we re-addressed this comparison while focusing on the more pressing, less-studied question for paediatric laboratories: paediatric samples are frequently low-volume and cytopenic, so the recommended 10–100 ng input is often unattainable, and runs must proceed with limited or compromised DNA to avoid delays in patient care. Because most of our assays use custom-designed amplicon panels, we retrospectively examined whether such sub-optimal input materially degrades data output and whether it can be reliably salvaged [12,13,14].

## 2. Materials and Methods

### 2.1. Study Design

This retrospective observational study curated NGS data from runs performed between February 2022 and July 2024. All consecutive clinical samples processed on custom amplicon panels during this period were eligible; a sample was included when both Nanoquant and pre-library Qubit readings and a completed sequencing run were available, and excluded when any of these records was missing. The 314 samples therefore constitute the complete laboratory cohort meeting these criteria, rather than a randomly drawn subset. Where a patient contributed more than one run, only the first qualifying run was retained. Our protocol uses custom amplicon-panel-based targeted DNA sequencing to detect germline pathogenic variants across a wide spectrum of paediatric disorders [15,16,17,18,19], using the Ion Torrent S5 GeneStudio platform (ThermoFisher Scientific, New York, NY, USA): DNA extraction, quantification and normalisation, library preparation, template generation and sequencing. The workflow and sample groups are summarised in Figure 1.

### 2.2. DNA Extraction and Quantification

EDTA blood was used for direct DNA isolation with the QIAamp DNA Blood Mini kit (Qiagen, Hilden, Germany) on the automated QIAcube platform. For low-volume samples (<1.5 mL) or low total leucocyte count (<5000/µL), manual extraction was performed from peripheral blood mononuclear cells isolated by density-gradient (Hypaque) separation. DNA was eluted in 100 µL. In line with the Ion AmpliSeq Library Kit Plus guide, library input is specified as total DNA mass per amplification reaction; each library used a fixed volume of input DNA per primer pool, so the Qubit concentrations reported here map to a defined total input mass per reaction. Specimen volume was not varied as an experimental factor, and the term “low-volume” refers to the pre-extraction sample rather than the library input. Quality and quantity were checked before storage on a Nanoquant Infinite 200 PRO (Tecan, Männedorf, Switzerland). Before library preparation, intact dsDNA was measured with the Qubit 1X dsDNA HS Assay on a Qubit 4 Fluorometer (Invitrogen/ThermoFisher, Waltham, MA, USA) per the manufacturer’s instructions.

### 2.3. Next-Generation Sequencing

Libraries were prepared with the Ion Ampliseq Library Plus kit and quantified using the Qubit 1X dsDNA HS Assay. Libraries were pooled at 100 pM and sequenced on Ion 520/530 chips on the Ion Torrent S5 sequencer. A mean depth ≥ 150× was used as the quality indicator for germline variant calling [20]. For inputs < 10 ng/µL, a standardised salvage protocol was used: additional target-amplification PCR cycles beyond the panel default, with an extended annealing/extension step, followed by loading of 1.5–2.0× the standard library volume at template generation. These adaptations were applied uniformly to low-input samples across the study period; because no contemporaneous standard-protocol control group of low-input samples was run, the success of low-input libraries cannot be attributed to these adaptations alone. Reads were analysed in Ion Reporter v5.16.2 (default settings): TMAP alignment to GRCh38.p2 or hg19 with custom-panel BED files, coordinate sorting, BAM conversion, duplicate removal (Picard) and germline calling with the Torrent Variant Caller. The 16 custom panels (ThermoFisher, Singapore) were designed in Ion AmpliSeq Designer with 400 bp chemistry and GRCh38 (three panels used 200 bp/hg19), span exons plus 10 bp flanks across two primer pools, and avoid repeat/high-GC regions where possible (Table 1).

### 2.4. Statistical Analysis

Continuous variables are reported as median (interquartile range, IQR). Paired Nanoquant–Qubit input was compared by the Wilcoxon signed-rank test, and their association by Spearman correlation. Agreement between methods was assessed by Bland–Altman analysis (linear bias and 95% limits of agreement, LoA; and log/ratio mean ratio with 95% LoA), with proportional bias tested by regressing the inter-method difference on the inter-method mean. Library quantification, mean depth and coverage QC parameters were compared between groups using the Mann–Whitney U test (exact test for *n* = 5 coverage subsets), with Cliff’s δ as the effect size. A linear mixed-effects model on log10(mean depth) with panel as a random intercept tested whether group depth differences persisted after accounting for panel composition. Library failure (post-library Qubit < 300 ng/mL) was modelled by univariable logistic regression and ROC analysis (Youden-optimal threshold). Plots used R 4.4.1 and Python 3 (SciPy, statsmodels, scikit-learn); GraphPad Prism 8.0.2 was used for descriptive plots. A two-sided *p* < 0.05 was significant.

## 3. Results

### 3.1. Cohort

During the study period, 314 samples were tested across 16 disease-specific panels (Table 1). The inherited bone marrow failure syndrome panel accounted for 43% (*n* = 136), followed by the paediatric metabolic and rare genetic disease panel (14%, *n* = 45) and the neuromuscular and complex-epilepsy panels (~8% each, *n* = 26 and 25). Amplicons per panel ranged from 86 to 2099 and genes from 4 to 130. All but three panels used GRCh38/400 bp chemistry. Mean turnaround time was 4–6 weeks.

### 3.2. DNA Quantification, Quality and Nanoquant–Qubit Agreement

Input DNA for all 314 samples was quantified by both methods (Figure 2A). Median concentrations were 59.75 ng/µL by Nanoquant (range 5.5–787) and 28.25 ng/µL by Qubit (range 1.82–550); the difference was highly significant (*p* < 0.0001; Figure 2B), and the median fold-difference was 2.12. Spearman correlation was positive and moderate (r = 0.531, *p* < 0.0001; Figure 2C and Table 2).

Crucially, correlation does not equal agreement. Bland–Altman analysis (Figure 3 and Table 2) showed a mean ratio of 2.17× but very wide 95% limits of agreement (0.57–8.33×), and a significant proportional bias (difference-versus-mean slope of 0.83, *p* < 0.0001); Nanoquant thus increasingly overestimated DNA at higher concentrations. Thus, although Nanoquant inflates readings ~2-fold on average, no single multiplicative correction factor can convert an individual Nanoquant value to its Qubit equivalent.

By Qubit input, 91.7% (288/314) of samples were Group A (≥10 ng/µL) and 8.3% (26/314) Group B (<10 ng/µL). The median A260/280 was 1.86 (range 1.48–2.37); 75.2% (236/314) fell within the ideal 1.7–1.9 window (Group C), while 24.8% (78/314) were outside it (Group D), of which 71 were >1.9 and 7 were <1.7 (Figure 2D,E).

### 3.3. Library Quantification, Sequencing Depth and Panel Adjustment

Post-library Qubit was significantly higher in Group A than Group B (median 1254 vs. 552 ng/mL; *p* < 0.0001; Cliff’s δ −0.47; Figure 4A; Table 3). Mean depth was, counter-intuitively, higher in Group B (1352× vs. 848×; unadjusted *p* = 0.025; Figure 4B). However, in a mixed-effects model accounting for sequencing panel, this difference was no longer significant (*p* = 0.083), indicating that the apparent depth advantage of low-input samples reflects panel/amplicon-number confounding and protocol adjustment (low-input libraries are loaded at 1.5–2.0× volume for template generation) rather than a true effect of low input. Library failure (<300 ng/mL) occurred in 19.2% (5/26) of Group B versus 3.1% (9/288) of Group A (Figure 4C,D).

For quality groups, input Qubit differed between Group C and D (median 30.1 vs. 21.1 ng/µL; *p* < 0.0001; Figure 4E). Post-library Qubit was modestly higher in Group C (1254 vs. 1026 ng/mL; *p* = 0.049), whereas mean depth was higher in Group D (1297× vs. 745×; *p* < 0.0001), a difference that persisted after panel adjustment (*p* = 0.001; Figure 4F). Effect sizes for all four group comparisons are summarised in Figure 5.

### 3.4. Prediction of Library Failure from Input DNA

Across the cohort, 14/314 (4.5%) libraries fell below the 300 ng/mL threshold. Input Qubit concentration discriminated failure with an ROC AUC of 0.79 (Figure 6); each 10-fold increase in input DNA reduced the odds of failure markedly (logistic OR 0.068 per log10 ng/µL; 95% CI 0.012–0.375; *p* = 0.002). The Youden-optimal input threshold was ≈21 ng/µL (sensitivity 0.93, specificity 0.68), approximately twice the manufacturer-recommended 10 ng/µL minimum (Table 4). Because this threshold was derived and evaluated in the same cohort with only 14 failure events and without independent internal validation, it should be regarded as exploratory and hypothesis-generating rather than a validated cut-off. Among the worst-case subset with both low quantity and low quality (Group B ∩ D, *n* = 13), only 3 (23%) failed; none of the 65 low-quality samples with adequate (>10 ng/µL) input failed.

### 3.5. Coverage Analysis

Five samples per group, selected by nearest-neighbour matching on post-library Qubit concentration so that coverage was compared at equivalent library yield (*n* = 20 total), underwent detailed coverage QC across ten parameters (percentage assigned amplicon reads, average reads per amplicon, uniformity of amplicon coverage, percentage base reads on target, average base coverage depth, target base coverage at 20× and 100×, average reads per primer pool 1 and 2, and amplicon reads (log10) versus %GC). None differed significantly between optimal and sub-optimal groups (all *p* > 0.20; Supplementary Tables S1 and S2), with small-to-moderate effect sizes only. With only five samples per group, these non-significant results indicate no detectable difference in this small subset rather than demonstrating equivalence of coverage. Both primer pools amplified comparably and no GC-related amplification bias was observed.

## 4. Discussion

Accurate DNA quantification underpins library preparation and sequencing output. Reframed around the practical question of whether sub-optimal paediatric DNA can be salvaged for amplicon NGS, this study delivers three actionable messages: fluorometry is non-negotiable but cannot be substituted by a correction factor; low-volume/compromised samples are usually recoverable with defined protocol adaptations; and an empirical input threshold (~20 ng/µL) better protects against library failure than the manufacturer’s minimum.

Qubit specifically measures intact dsDNA [21,22,23], avoiding overestimation from RNA, single-stranded or fragmented DNA, protein, and free nucleotides [11]. Consistent with prior reports of 3- to 5-fold spectrophotometric inflation [7,8,9,10] and with Masago et al.’s NanoDrop-versus-Qubit comparison in lung cancer specimens [3], Nanoquant overestimated DNA ~2.1-fold. Our novel contribution is the agreement analysis: the wide, concentration-dependent Bland–Altman limits show that the 2-fold inflation is an average, not a usable constant, a clinically important caution for laboratories tempted to ‘correct’ spectrophotometric values rather than re-measure on a fluorometer [24,25].

Few studies address low-volume blood with compromised quality/quantity, as opposed to FFPE tissue [26,27]. Using a 10 ng/µL input cut-off, Group A had higher post-library yield than Group B (1254 vs. 552 ng/mL; *p* < 0.0001), yet Group B libraries still exceeded the 300 ng/mL template-generation threshold in 81% of cases. This is attributable to our standardised salvage protocol: increased PCR cycle numbers and extension times for <10 ng/µL inputs, and a 1.5–2.0× library input volume for template generation. The paradoxically higher mean depth in Group B, which the data alone might suggest is a real benefit, was shown by mixed-effects modelling to be confounding by panel composition (*p* = 0.083 after adjustment), compounded by the higher loading volume.

DNA quality groups behaved similarly: high-quality Group C marginally outperformed Group D on library yield, while both achieved comparable input and Group D showed higher depth (persisting after adjustment, likely reflecting panel mix). The robustness of low-quality samples is plausibly due to the triple AMPure bead clean-up during library preparation, which removes salts, protein and unwanted fragments [28]. A260/280 reflects sample purity rather than DNA integrity; a value outside 1.7–1.9 flags possible contamination but does not by itself establish degradation, and our use of “compromised” should be read in this narrower, purity-based sense. Only 23% of the double-compromised subset (3/13) failed library QC, and no low-quality sample with adequate input failed.

The ROC-derived input threshold of ~21 ng/µL is a practical, data-driven refinement: it captured 93% of failures and indicates that the manufacturer’s 10 ng/µL is permissive for compromised paediatric material [29]. Finally, an extended ten-parameter coverage analysis found no significant differences between groups, indicating that while input quantity and quality affect library yield and (apparently) depth, they do not degrade core coverage performance; most custom panels amplified evenly across both pools and GC-rich regions [30].

Limitations include the retrospective design and potential selection bias inherent to a single-centre laboratory cohort, the small Group B (*n* = 26) and the underpowered *n* = 5 coverage subsets, which preclude formal equivalence testing. The five coverage-QC samples per group were selected by post-library concentration matching rather than at random, so these subset comparisons are descriptive and cannot establish equivalent coverage.

## 5. Conclusions

Qubit quality control is strongly recommended before amplicon sequencing, and DNA normalisation should use Qubit rather than Nanoquant/NanoDrop, which inflate readings by ~2.1-fold and lack a reliable correction factor. Low-volume or compromised paediatric DNA (<10 ng/µL and/or A260/280 outside 1.7–1.9) yields lower library concentration and a higher library-failure rate but can frequently be salvaged for post-library quantification, sequencing depth or coverage QC when salvage steps (additional PCR cycles/extension time, or 1.5–2.0× library input volume) are applied; only ~19–23% of the worst-case samples fail library QC. An exploratory, single-cohort empirical input threshold of ≈20 ng/µL is associated with the lowest risk of library failure in this dataset, and most custom amplicon panels perform robustly across both primer pools and GC-rich regions.

## Figures and Tables

**Figure 1:**
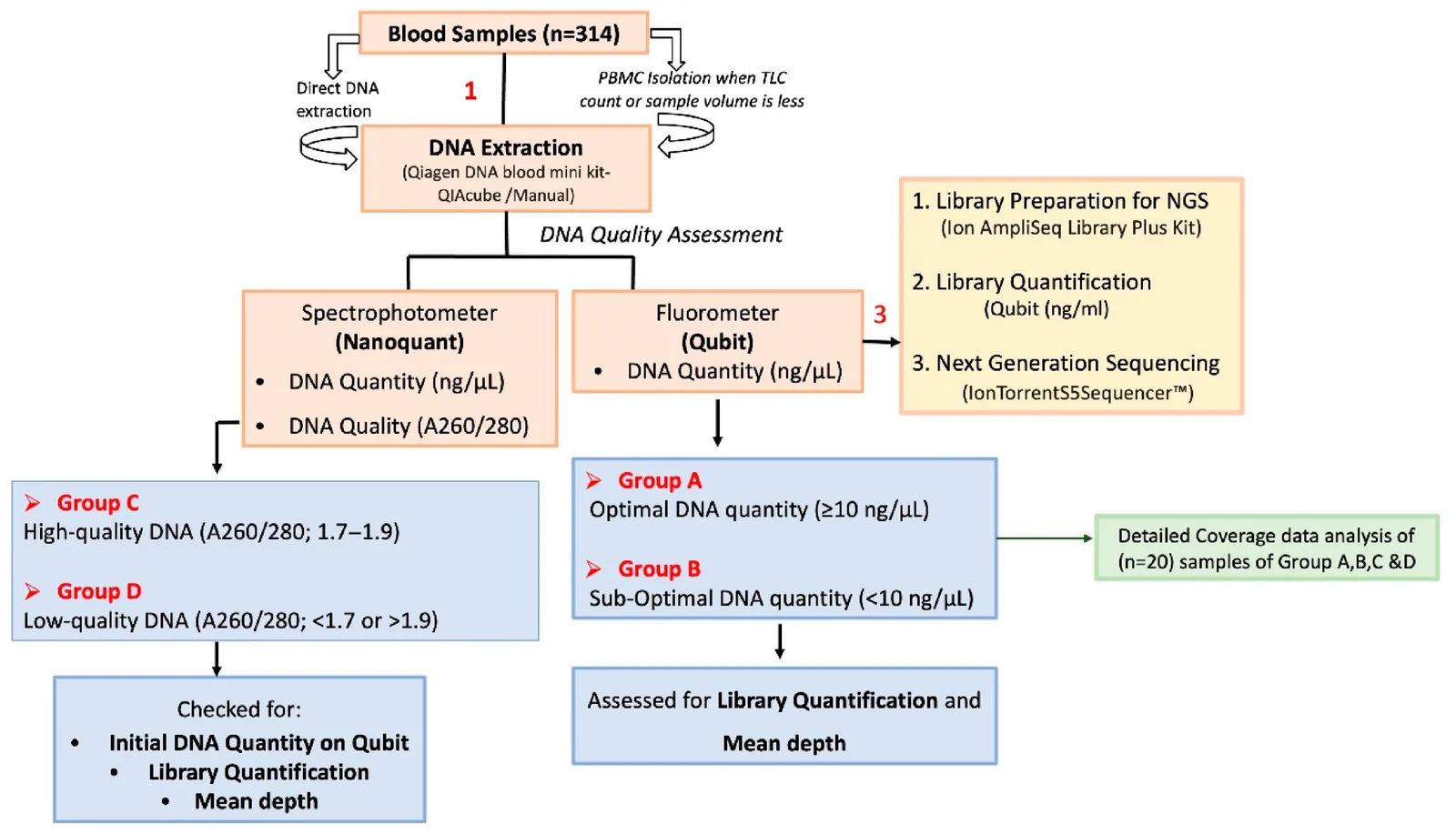
Study workflow and sample groups. Schematic of the 314-sample amplicon-NGS pipeline, showing DNA extraction, dual quantification (Nanoquant, Qubit), grouping by input quantity (A/B) and quality (C/D), and the 20-sample coverage QC subset.

**Figure 2:**
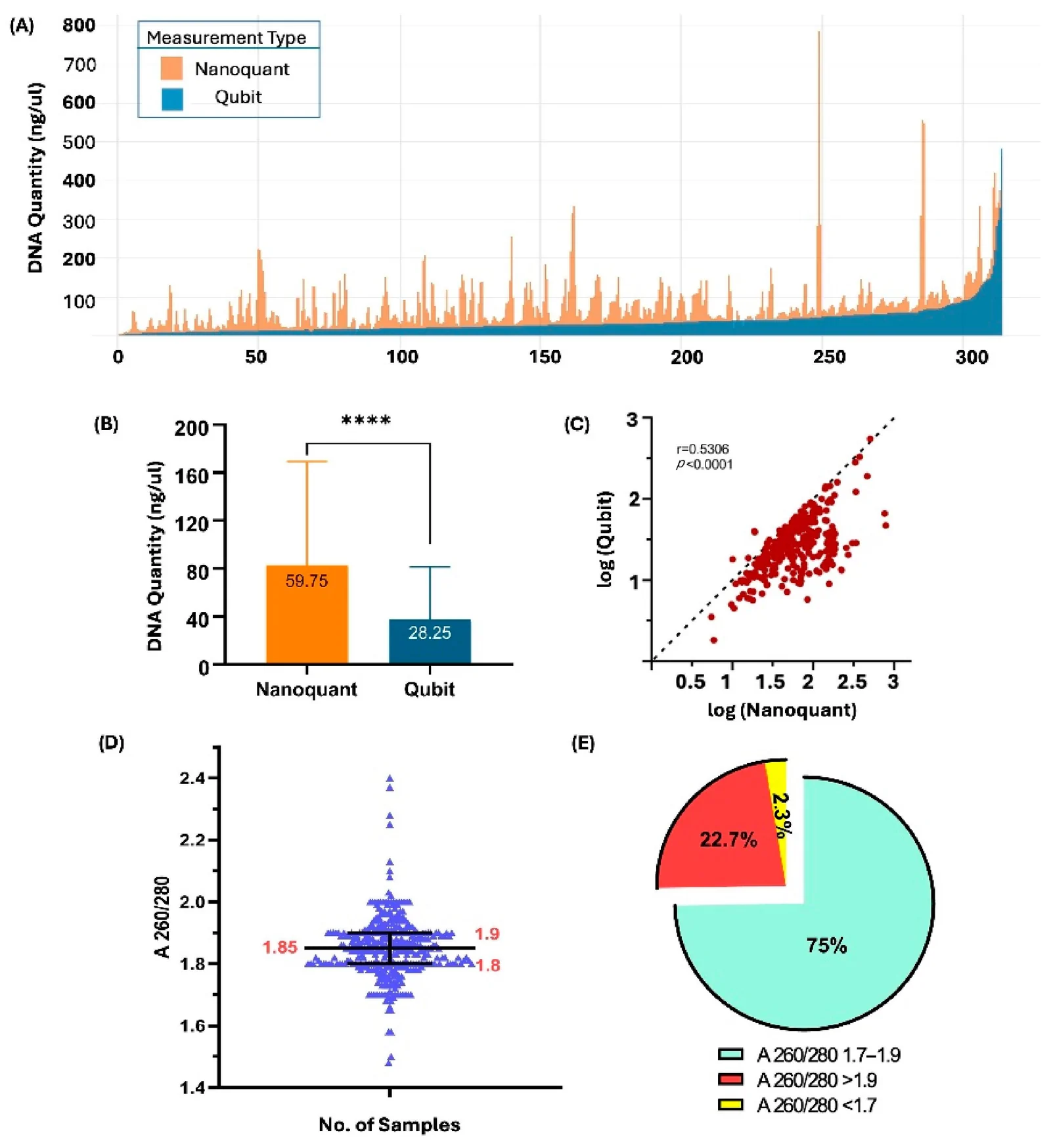
DNA quantification and quality. (**A**) Per-sample Nanoquant and Qubit input concentrations. (**B**) Median Nanoquant (59.75 ng/µL) versus Qubit (28.25 ng/µL); *p* < 0.0001. (**C**) Spearman correlation of log-transformed readings (r = 0.531). (**D**) Distribution of A260/280 (median 1.86). (**E**) Proportion of samples with A260/280 1.7–1.9 (75.2%), >1.9 (22.6%) and <1.7 (2.2%). **** - significant *p*-value.

**Figure 3:**
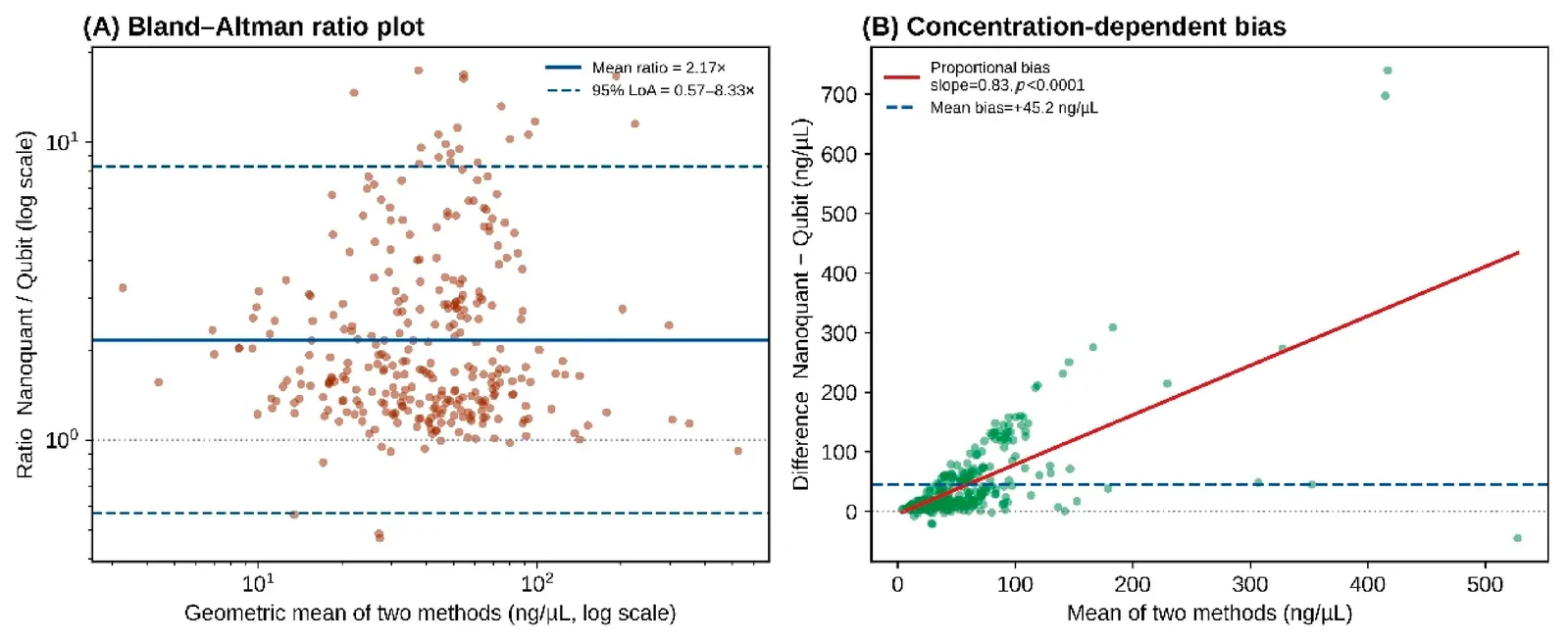
Bland–Altman agreement between Nanoquant and Qubit. (**A**) Ratio plot: mean ratio 2.17× with wide 95% limits of agreement (0.57–8.33×). (**B**) Difference-versus-mean plot showing significant proportional bias (slope 0.83, *p* < 0.0001)—overestimation increases with concentration.

**Figure 4:**
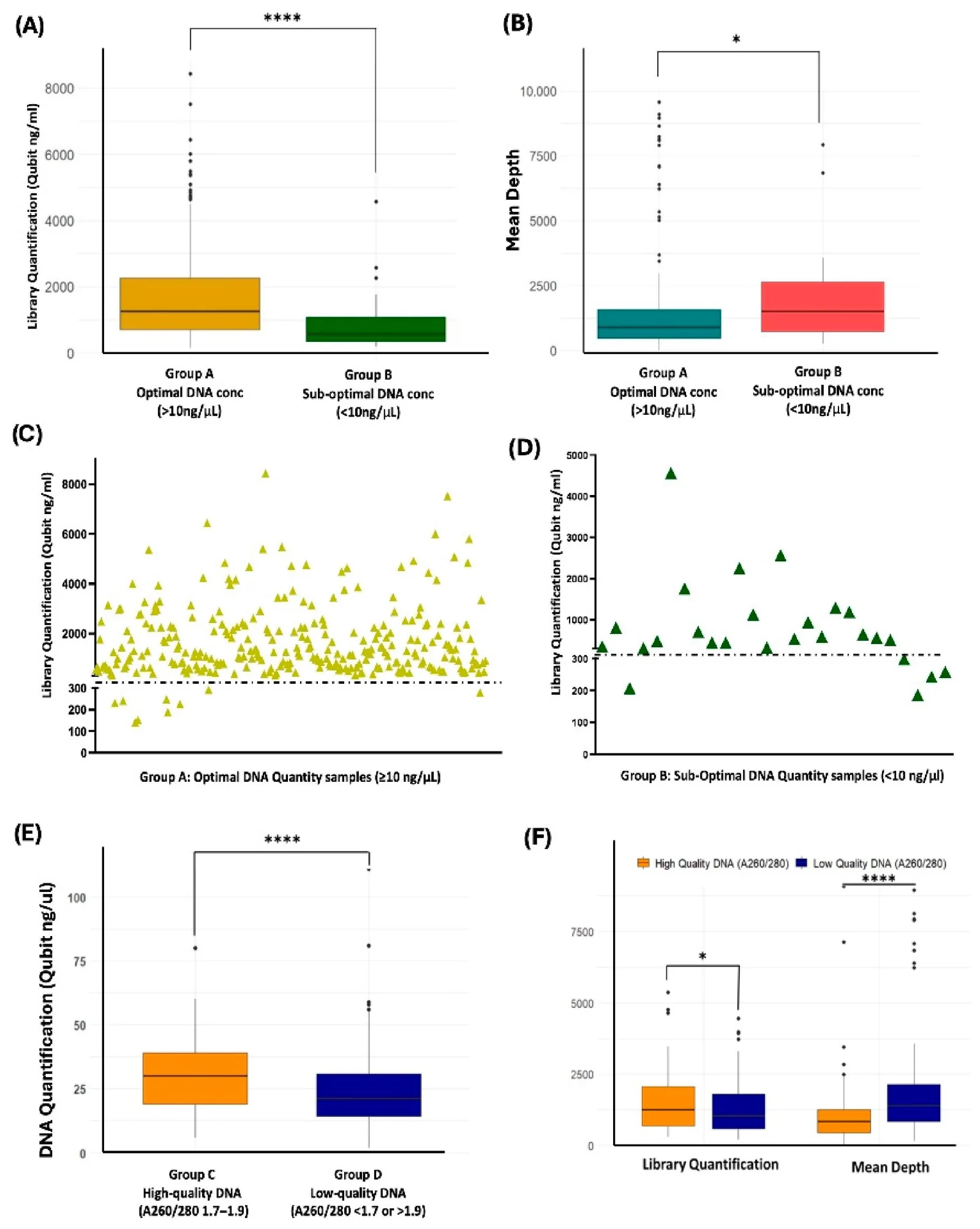
Library quantification and sequencing depth by group. (**A**) Library quantification A versus B. (**B**) Mean depth A versus B. (**C**,**D**) Library quantification scatter for Groups A and B (300 ng/mL threshold dashed). (**E**) Input Qubit for high- versus low-quality DNA (C vs. D). (**F**) Library quantification and mean depth for quality groups. *, **** - significant *p*-value.

**Figure 5:**
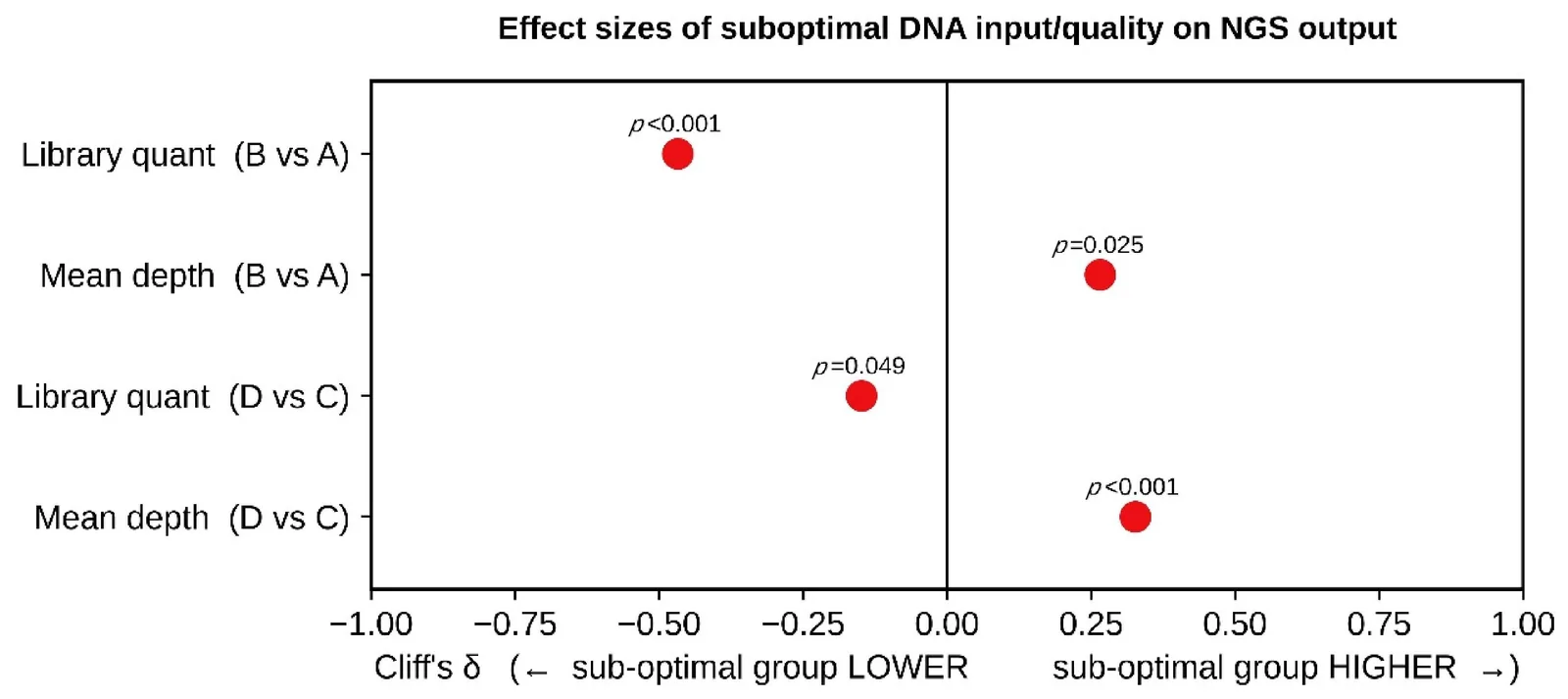
Effect sizes of sub-optimal DNA input/quality on NGS output. Cliff’s δ for the four group comparisons (negative = sub-optimal group lower; positive = sub-optimal group higher): sub-optimal samples yield lower library concentration but higher mean depth.

**Figure 6:**
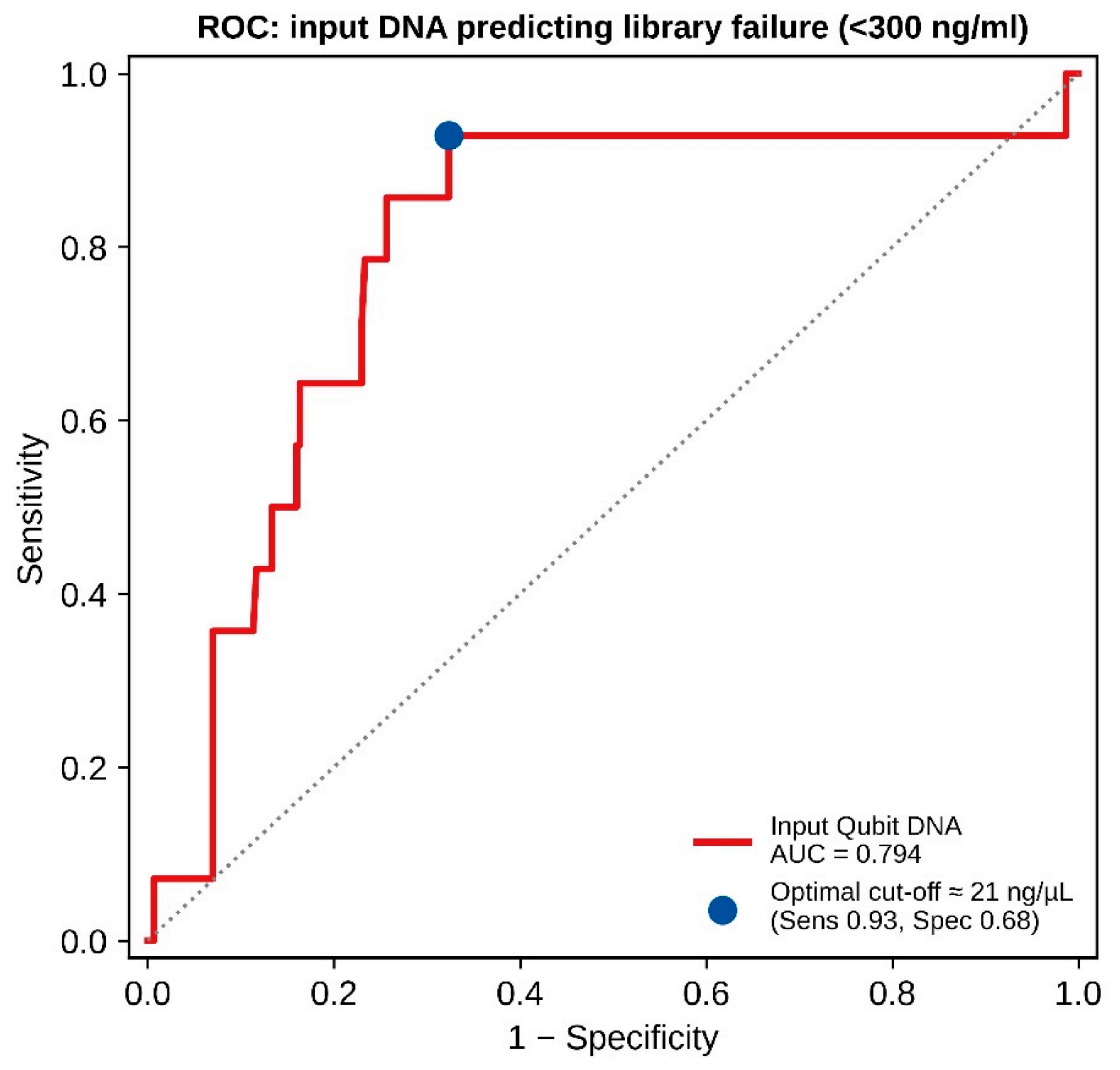
ROC analysis for prediction of library failure. Input Qubit DNA concentration discriminates post-library failure (<300 ng/mL) with AUC 0.79; the Youden-optimal threshold is ≈21 ng/µL (sensitivity 0.93, specificity 0.68).

**Table 1:** Custom-designed amplicon panels with their IAD numbers, gene/amplicon content and number of cases processed.

S. No.	Panel Name	IAD No.	Genes Targeted	Amplicons	Reference Genome	Samples (*n*)
1	Inherited bone marrow failure syndromes (IBMFS)	151,910	33	494	GRCh38	136
2	Mendelian susceptibility to mycobacterial disease	205,833	33	613	GRCh38	4
3	Primary immunodeficiency disorder	150,236	44	672	GRCh38	22
4	Complex childhood epilepsy	193,075	15	368	GRCh38	25
5	Leukodystrophies	208,472	23	312	GRCh38	7
6	Iron metabolism defects	202,329	26	297	GRCh38	14
7	Paediatric metabolic and rare genetic disorders	191,968	30	466	hg19	45
8	Neuromuscular disorders	207,233	19	584	GRCh38	26
9	Haemolytic anaemias	194,951	33	721	GRCh38	1
10	Hemophagocytic lymphohistiocytosis	202,233	7	86	GRCh38	13
11	Mitochondrial disorders	207,408	130	2099	hg19	1
12	Paediatric autoimmune defects	224,476	14	356	GRCh38	7
13	Severe combined immunodeficiency	227,519	15	343	GRCh38	6
14	Neurodegeneration with brain iron accumulation	217,698	18	460	GRCh38	3
15	Neuronal ceroid lipofuscinosis	217,696	17	354	hg19	1
16	Tuberous sclerosis complex	232,960	4	238	GRCh38	3

IAD, Ion AmpliSeq Designer. Panels 7, 11 and 15 were designed on hg19/200 bp chemistry; the remainder used GRCh38/400 bp chemistry. Total = 314 samples.

**Table 2:** Comparison and agreement between spectrophotometric (Nanoquant) and fluorometric (Qubit) DNA quantification (*n* = 314).

Parameter	Nanoquant (Spectrophotometry)	Qubit (Fluorometry)
Median DNA, ng/µL (IQR)	59.75 (36.2–96.4)	28.25 (16.8–42.0)
Range, ng/µL	5.5–787	1.82–550
Median fold-difference (Nanoquant/Qubit)	2.12×	
Wilcoxon signed-rank (paired)	*p* < 0.0001	
Spearman correlation	r = 0.531; *p* < 0.0001	
Bland–Altman mean bias (linear)	+45.2 ng/µL; 95% LoA −101 to +191	
Bland–Altman mean ratio (log)	2.17×; 95% LoA 0.57–8.33×	
Proportional bias (difference vs. mean)	slope 0.83; *p* < 0.0001	

IQR, interquartile range; LoA, limits of agreement. Nanoquant systematically overestimates DNA ~2.1-fold, but the wide, concentration-dependent limits of agreement indicate that a single constant correction factor cannot reliably convert Nanoquant values to their Qubit equivalents at the individual-sample level.

**Table 3:** Effect of sub-optimal DNA quantity (Group A vs. B) and quality (Group C vs. D) on library quantification and sequencing depth, with effect sizes.

Comparison/Outcome	Group (Optimal) Median (IQR)	Group (Sub-Optimal) Median (IQR)	*p* (Mann–Whitney)	Cliff’s δ *	Panel-Adjusted *p* †
Quantity—Library quant (ng/mL)	A: 1254 (687–2254)	B: 552 (332–1085)	<0.0001	−0.47	—
Quantity—Mean depth (×)	A: 848 (423–1385)	B: 1352 (552–2254)	0.025	+0.27	0.083 (NS)
Quality—Input Qubit (ng/µL)	C: 30.1 (18.9–46.0)	D: 21.1 (14.3–29.8)	<0.0001	−0.30	—
Quality—Library quant (ng/mL)	C: 1254 (668–2318)	D: 1026 (579–1820)	0.049	−0.15	—
Quality—Mean depth (×)	C: 745 (392–1270)	D: 1297 (790–1699)	<0.0001	+0.33	0.001

* Cliff’s δ orientation: negative = sub-optimal group lower; positive = sub-optimal group higher. † Linear mixed-effects model on log10 depth with sequencing panel as a random intercept. The unadjusted higher depth in quantity Group B loses significance after adjusting for panel (*p* = 0.083), indicating confounding by panel/amplicon number rather than a true input effect; the quality-group depth difference persists. NS, not significant.

**Table 4:** Post-library quantification failure (<300 ng/mL) by group and prediction of failure from input DNA.

Group	Failures/*n*	Failure Rate	Notes
A—Optimal quantity (≥10 ng/µL)	9/288	3.1%	
B—Sub-optimal quantity (<10 ng/µL)	5/26	19.2%	
C—High quality (A260/280 1.7–1.9)	11/236	4.7%	
D—Low quality (A260/280 <1.7 or >1.9)	3/78	3.8%	
Low quantity AND low quality (B ∩ D)	3/13	23.1%	worst-case subset
Whole cohort	14/314	4.5%	

Prediction of library failure from input Qubit DNA: ROC AUC = 0.79; logistic OR = 0.068 per log10 ng/µL (95% CI 0.012–0.375; *p* = 0.002). Youden-optimal input threshold ≈ 21 ng/µL (sensitivity 0.93, specificity 0.68)—approximately double the manufacturer-recommended 10 ng/µL minimum. Failure defined as post-library Qubit < 300 ng/mL (template-generation QC threshold). Estimates from 14 failure events should be regarded as exploratory (rare-event analysis).

## Data Availability

The original contributions presented in this study are included in the Supplementary Materials. Further inquiries can be directed to the corresponding author.

## Supplementary Materials

The following supporting information can be downloaded at: https://www.mdpi.com/article/10.3390/diagnostics16213447/s1, Supplementary Table S1: Coverage QC metrics in five representative samples per Group- DNA quantity groups (A vs B), Supplementary Table S2: Coverage QC metrics in five representative samples per Group- DNA quality groups (C vs D), Supplementary Data S1: Raw NGS data for DNA quantification study (n = 314).

## References

[1] Bruijns B., Hoekema T., Oomens L., Tiggelaar R., Gardeniers H. (2022). Performance of spectrophotometric and fluorometric DNA quantification methods. Analytica.

[2] Sang F., Ren J. (2006). Comparisons between capillary zone electrophoresis and real-time PCR for quantification of circulating DNA levels in human sera. J. Chromatogr. B.

[3] Masago K., Fujita S., Oya Y., Takahashi Y., Matsushita H., Sasaki E., Kuroda H. (2021). Comparison between fluorimetry (Qubit) and spectrophotometry (NanoDrop) in the quantification of DNA and RNA extracted from frozen and FFPE tissues from lung cancer patients. Medicina.

[4] Garcia-Elias A., Alloza L., Puigdecanet E., Nonell L., Tajes M., Curado J., Enjuanes C., Díaz O., Bruguera J., Martí-Almor J. (2017). Defining quantification methods and optimizing protocols for microarray hybridization of circulating microRNAs. Sci. Rep..

[5] Klein D. (2002). Quantification using real-time PCR technology: Applications and limitations. Trends Mol. Med..

[6] Pedini P., Graiet H., Laget L., Filosa L., Chatron J., Cherouat N., Chiaroni J., Hubert L., Frassati C., Picard C. (2021). Qualitative and quantitative comparison of cell-free DNA and cell-free fetal DNA isolation by four (semi-)automated extraction methods. J. Transl. Med..

[7] Simbolo M., Gottardi M., Corbo V., Fassan M., Mafficini A., Malpeli G., Lawlor R.T., Scarpa A. (2013). DNA qualification workflow for next generation sequencing of histopathological samples. PLoS ONE.

[8] Heydt C., Fassunke J., Künstlinger H., Ihle M.A., König K., Heukamp L.C., Schildhaus H.U., Odenthal M., Büttner R., Merkelbach-Bruse S. (2014). Comparison of pre-analytical FFPE sample preparation methods and their impact on massively parallel sequencing in routine diagnostics. PLoS ONE.

[9] Sharma P., Singh M., Singh A., Bhardwaj D., Bhatia P. (2022). Experience of quantity and quality of DNA and RNA extraction from limited pediatric blood samples: A comparative analysis of automated and manual kit-based method. Indian J. Pathol. Microbiol..

[10] Kapp J.R., Diss T., Spicer J., Gandy M., Schrijver I., Jennings L.J., Li M.M., Tsongalis G.J., de Castro D.G., Bridge J.A. (2015). Variation in pre-PCR processing of FFPE samples leads to discrepancies in BRAF and EGFR mutation detection: A diagnostic RING trial. J. Clin. Pathol..

[11] Versmessen N., Van Simaey L., Negash A.A., Vandekerckhove M., Hulpiau P., Vaneechoutte M., Cools P. (2024). Comparison of DeNovix, NanoDrop and Qubit for DNA quantification and impurity detection of bacterial DNA extracts. PLoS ONE.

[12] McNulty S.N., Mann P.R., Robinson J.A., Duncavage E.J., Pfeifer J.D. (2020). Impact of reducing DNA input on next-generation sequencing library complexity and variant detection. J. Mol. Diagn..

[13] Abul-Husn N.S., Marathe P.N., Kelly N.R., Bonini K.E., Sebastin M., Odgis J.A., Abhyankar A., Brown K., Di Biase M., Gallagher K.M. (2023). Molecular diagnostic yield of genome sequencing versus targeted gene panel testing in racially and ethnically diverse pediatric patients. Genet. Med..

[14] Sánchez Suárez A., Martínez Menéndez B., Escolar Escamilla E., Martínez Sarries F.J., Esparza Garrido M.I., Gil-Fournier B., Ramiro León S., Rubio Gribble B., Quesada Espinosa J.F., Alcaraz Romero A.J. (2024). Whole exome sequencing and panel-based analysis in 176 Spanish children with neurodevelopmental disorders. Genes.

[15] Schneider K.W., Suttman A., McKinney C., Giller R., Dollerschell K., Nakano T.A. (2022). Incorporating genetic counseling into the evaluation of pediatric bone marrow failure. J. Genet. Couns..

[16] Baxter S.K., Walsh T., Casadei S., Eckert M.M., Allenspach E.J., Hagin D., Segundo G., Lee M.K., Gulsuner S., Shirts B.H. (2022). Molecular diagnosis of childhood immune dysregulation, polyendocrinopathy, and enteropathy, and implications for clinical management. J. Allergy Clin. Immunol..

[17] Mir A., AlQahtani M., Amer F., AlBaradie R., AlOtaibi W., AlGhamdi F., Khallaf H., Bashir S., Costain G., Aljouda L. (2024). Diagnostic evaluation of patients with epileptic spasms in the era of next-generation sequencing. Epileptic Disord..

[18] Kulsirichawaroj P., Chanvanichtrakool M., Wattanadilokchatkun P., Pho-Iam T., Limwongse C., Likasitwattanakul S., Boonyapisit K., Sanmaneechai O., Nishino I., Shotelersuk V. (2025). Next-generation sequencing for pediatric-onset neuromuscular disorders unresolved by conventional diagnostic methods. Pediatr. Res..

[19] Simões M., Santos M.J., Martins S., Macário M.D.C., Durães J., Diogo L., Oliveira J.P., Ferreira J.C., Grazina M. (2024). Challenges in genetic diagnosis of mitochondrial diseases: What can functional genomics’ studies do?. Endocr. Metab. Immune Disord. Drug Targets.

[20] Yan H., Mo Y., Liu S., Luo X., Liu L., Zhou L., Zhang X., Chen Y., Cao K. (2022). Hemophagocytic lymphohistiocytosis in a child with primary immunodeficiency infected with Talaromyces marneffei. Front. Immunol..

[21] Paul P., Rajput S., Joshi P., Naithani M., Chowdhury N., Rao S., Pai M.O. (2021). Comparison of fluorometric and UV spectrophotometric findings for DNA isolated from formalin-fixed paraffin-embedded blocks, fine-needle aspiration cytology smears, and blood. Cureus.

[22] Khetan D., Gupta N., Chaudhary R., Shukla J.S. (2019). Comparison of UV spectrometry and fluorometry-based methods for quantification of cell-free DNA in red cell components. Asian J. Transfus. Sci..

[23] Lee Y.-H., Tsai C.-Y., Lu Y.-S., Lin P.-H., Chiang Y.-T., Yang T.-H., Hsu J.S.-J., Hsu C.-J., Chen P.-L., Liu T.-C. (2023). Revisiting genetic epidemiology with a refined targeted gene panel for hereditary hearing impairment in the Taiwanese population. Genes.

[24] Gerke O. (2020). Reporting standards for a Bland-Altman agreement analysis: A review of methodological reviews. Diagnostics.

[25] Taffé P. (2021). When can the Bland & Altman limits of agreement method be used and when it should not be used. J. Clin. Epidemiol..

[26] Kim S.M., Roh S.K., Ham J.Y., Kim Y.K., Chang S.H. (2025). Clinical, immunologic, and genetic characteristics of T-lymphoblastic leukemia with STIL-TAL1 fusion. Clin. Lab..

[27] Shatalov P.A., Raygorodskaya M.P., Shinkarkina A.P., Traspov A.A., Murzaeva A.V., Doroshenko Y.A., Leuhina I.A., Mileyko V.A., Ivanov M.V., Grigoreva T.V. (2025). Adaptation of PCR-based library preparation for the MGI platform for cancer mutation testing in a clinical setting. PLoS ONE.

[28] Sharma A., Kumar A., Rawat K., Vij S., Sandhu A., Gautam V., Saha P.K., Saha L. (2024). Novel TMPRSS6 variants and their impact on iron-refractory iron deficiency anaemia in pregnancy: A North Indian genotype-phenotype study. Br. J. Haematol..

[29] Al-Jamea L.H., Woodman A., MHeiba N., Elshazly S.A., Ben Khalaf N., Al-Yami F.S., Bilal Waheed K., Al Mutair A., Alsedi A., Quiambao J.V. (2023). TMPRSS6 gene mutations in six Saudi families with iron-refractory iron deficiency anemia. Gene.

[30] Cay E., Sezer A., Karakulak V., Serbes M., Ozcan D., Bisgin A., Aygunes U., Sasmaz H.I., Yucel S.P., Toyran T. (2023). Hemophagocytic lymphohistiocytosis in children with Griscelli syndrome type 2: Genetics, laboratory findings and treatment. Am. J. Clin. Exp. Immunol..

