# Supplementary material for: Good Enough to Sequence: Fluorometry-Guided Salvage of Low-Volume and Compromised Paediatric DNA in Custom Amplicon Next-Generation Sequencing
Source: Diagnostics (Basel). 2026 Oct 22;16(21):3447. doi: 10.3390/diagnostics16213447 (PMC13762391; doi:10.3390/diagnostics16213447)
Supplement: Supplementary file 1 [file diagnostics-16-03447-s001.zip › diagnostics-4399515-supplementary.pdf]

SUPPLEMENTARY MATERIAL

Fluorometry-guided salvage of low-volume and compromised paediatric DNA in custom amplicon next-generation sequencing

Supplementary Table S1. Coverage QC metrics in five representative samples per group — DNA quantity groups (A vs. B).

Coverage QC parameter	Optimal mean	Sub-optimal mean	Cliff's δ	p
Percentage Assigned Amplicon Read	95.73	95.91	-0.28	0.5476
Average Read Per Amplicon	872.8	1686.4	-0.28	0.5476
Uniformity Of Amplicon Coverage	95	95.31	-0.52	0.2087
Percentage Base Reads On Targets	95.17	95.44	-0.28	0.5476
Average Base Coverage Depth	820	1605.08	-0.24	0.6004
Target Base Coverage At 20X	98.42	98.43	-0.08	0.9166
Target Base Coverage At 100X	88.36	93.71	-0.2	0.6905
Avg. Amplicon Read Primer Pool 1	612	1446	-0.28	0.5476
Avg. Amplicon Read Primer Pool 2	646	1438	-0.28	0.5476
No. of Amplicon Reads (log10) vs. %GC	2.78	2.98	-0.28	0.5271

Means of n = 5 samples per group; two-sided exact Mann–Whitney U test. None of the parameters differed significantly (all p > 0.20). Cliff's δ orientation: negative = sub-optimal (B) lower. The 'amplicons with ≥20 reads' row from the original draft was excluded because its entries mixed percentages (94–99) with absolute amplicon counts (≈298–300), making the row mean uninterpretable; this field requires source correction before inclusion.

Supplementary Table S2. Coverage QC metrics in five representative samples per group — DNA quality groups (C vs. D).

Coverage QC parameter	Optimal mean	Sub-optimal mean	Cliff's δ	p
Percentage Assigned Amplicon Read	95.53	95.61	-0.04	1
Average Read Per Amplicon	658.8	787	-0.28	0.5476
Uniformity of Amplicon Coverage	94.02	95.08	-0.52	0.2045
Percentage Base Reads On Targets	95.04	94.98	-0.12	0.834
Average Base Coverage Depth	971	854.6	0.12	0.8413
Target Base Coverage At 20X	98.42	98.36	-0.08	0.9166
Target Base Coverage At 100X	86.9	80.78	0.36	0.4206
Avg. Amplicon Read Primer Pool 1	896	860	-0.12	0.8335
Avg. Amplicon Read Primer Pool 2	924	926	-0.04	1
No. of Amplicon Reads (log10) vs. %GC	2.76	2.81	-0.12	0.8335

Means of n = 5 samples per group; two-sided exact Mann–Whitney U test. None of the parameters differed significantly (all p > 0.20). Given n = 5 per arm, these comparisons are exploratory and underpowered; the consistent absence of large effect sizes is supportive of, but not formal proof of, equivalence.

Supplementary Methods — Statistical addendum

Continuous variables are summarised as median (interquartile range, IQR). Paired comparison of Nanoquant and Qubit input concentrations used the Wilcoxon signed-rank test; their association used the Spearman rank correlation. Agreement between the two quantification methods was assessed by Bland–Altman analysis on both the linear scale (mean bias and 95% limits of agreement, LoA) and the log10/ratio scale (mean ratio and 95% LoA), and proportional bias was tested by regressing the inter-method difference on the inter-method mean.

Group comparisons (quantity A vs. B; quality C vs. D) for library quantification, mean sequencing depth, and coverage QC parameters used the Mann–Whitney U test (exact test for the n = 5 coverage subsets), with Cliff's δ reported as a non-parametric effect size. To determine whether the higher mean depth observed in sub-optimal

groups was an artefact of differing panel composition, a linear mixed-effects model was fitted to log10(mean depth) with group as a fixed effect and sequencing panel as a random intercept.

Prediction of post-library failure (Qubit < 300 ng/ml) from input DNA concentration was evaluated by univariable logistic regression (odds ratio per log10 ng/µL) and a receiver operating characteristic (ROC) analysis; the Youden index identified the optimal input threshold. As only 14 failure events were observed, these estimates are exploratory. Analyses were performed in Python 3 (scipy 1.17, statsmodels 0.14, scikit-learn 1.8); a two-sided $p < 0.05$ was considered significant.

Supplementary Note — Dataset integrity

During re-analysis, the secondary worksheet of the source dataset was found to have its Qubit-concentration column independently sorted, which dissociated the per-sample correspondence between columns (cross-column sample match rate < 1%). That worksheet is therefore valid only for column-wise marginal summaries (medians, ranges, counts) and not for any paired, correlation, regression or per-sample analysis. The primary worksheet was intact and row-aligned (reproducing the reported Nanoquant–Qubit Spearman r = 0.531) and was used for all analyses. A cleaned, analysis-ready version of the dataset is provided as Supplementary Data S1.
